# Clinical Outcome and Biomechanical Analysis of Dynamic Hip Screw Combined with Derotation Screw in Treating Displaced Femoral Neck Fractures Based on Different Reduction Qualities in Young Patients (≤65 Years of Age)

**DOI:** 10.1155/2022/9505667

**Published:** 2022-01-05

**Authors:** Jian Zhu, Yonglong Li, Yingze Zhang, Xiaodong Cheng

**Affiliations:** ^1^School of Medicine, Nankai University, Tianjin 300071, China; ^2^Shanxi Bethune Hospital, Shanxi Academy of Medical Science, No. 99, Longcheng Street, Taiyuan, 030032 Shanxi Province, China; ^3^Department of Orthopaedic Surgery, The Third Hospital of Hebei Medical University, Shijiazhuang 050051, China; ^4^Key Laboratory of Biomechanics of Hebei Province, Orthopaedic Research Institution of Hebei Province, Shijiazhuang, 050051 Hebei, China; ^5^NHC Key Laboratory of Intelligent Orthopeadic Equipment (The Third Hospital of Hebei Medical University), Shijiazhuang 050051 Hebei, China

## Abstract

**Objective:**

To examine the clinical results and biomechanical mechanism of the dynamic hip screw (DHS) and derotation screw (DS) in the treatment of displaced femoral neck fractures (FNF) based on different reduction qualities in young patients (≤65 years of age).

**Methods:**

All patients with FNF who received closed reduction and internal fixation with DHS+DS from January 2014 to August 2019 were retrospectively analyzed. Data on demographics, surgery, clinical outcomes, and postoperative complications were collected. According to the reduction quality immediately after surgery, all patients were categorized into the positive buttress reduction group (PBRG) and the anatomical reduction group (ARG). The complications and clinical outcomes were compared between the two groups. Meanwhile, the biomechanical mechanism of different reduction qualities was further analyzed with finite element analysis (FEA). The distribution of von Mises stress, the peak stress of internal fixation, and the displacement of the proximal fragment were compared between the two groups.

**Results:**

A total of 68 patients were included in our study. Among them, 31 were divided into the PBRG while 37 were in the ARG. The surgical time and fluoroscopy time were significantly shorter in the PBRG than in the ARG (*p* < 0.05). The degree of femoral neck shortening and the varus change of the femoral-neck shaft angle were lower in the PBRG compared to the ARG (*p* < 0.05). The excellent-good rate of the Harris hip score was higher in the PBRG compared to the ARG (83.9% vs. 64.8%). The FEA results demonstrated that the stress of DHS+CS and the downward displacement of the proximal femoral neck fragment were greater in the ARG than in the PBRG.

**Conclusion:**

For displaced FNF with difficulty to achieve reduction, DHS+CS combined with positive buttress reduction was an effective treatment in young patients due to better mechanical support, shorter surgical time, less radiation exposure, and higher excellent-good rate of Harris hip score.

## 1. Introduction

Femoral neck fractures (FNFs) are commonly seen injuries and account for nearly half of proximal femoral fractures [1]. Most of this injury requires an operation to prevent bed-related complications. For young FNF patients, reduction and internal fixation is the preferred treatment method with the advantage of preservation of the native femoral head [2, 3]. Among internal fixation implants, the dynamic hip screw (DHS) combined with the derotational screw (DS) is widely used [4–6]. Compared to the cannulated screw system (CCS), DHS+DS could provide better resistance to shear and rotation forces [7, 8]. In a biomechanical study, Freitas et al. [9] reported that DHS+DS may reduce nearly 15% of vertical displacement and more than 50% of rotational displacement compared with CCS.

During the treatment procedure of displaced fractures, reduction quality plays an important role in the stability of fracture fixation. Although the primary goal of treating fractures is anatomical reduction, it is sometimes difficult to achieve, especially in displaced FNF [10, 11]. Multiple attempts of closed reduction or open reduction with the aim of anatomical reduction can damage the blood supply of the femoral head and increase the incidence of fracture nonunion or necrosis of the femoral head [12]. Recently, nonanatomical reductions of FNF, which include positive buttress reduction and negative buttress reduction, have attracted wide attention [13–15]. The definition of the former is that the distal fracture segments is positioned medially to the lower-medial edge of the proximal segment, while the latter is that the proximal fracture segment is positioned medially to the upper medial edge of the distal segment [16]. Since Gotfried et al. first proposed the concept of nonanatomical reduction in 2013, numerous studies have compared the effect of nonanatomical reduction and anatomical reduction in FNF [10, 13–15]. However, all of these studies focused on internal fixation with CCS. The data on the effect of DHS+DS in different reduction qualities of FNFs remains scant. Moreover, to our knowledge, there was no study that analyzed the effect of different reduction qualities after DHS+DS fixation from the perspective of biomechanical mechanism.

It is well known that negative buttress reduction is associated with a high rate of internal fixation failure and should be avoided [16–18]. Therefore, the objective of this study was to compare the positive buttress reduction with anatomical reduction of displaced FNFs in young patients which received closed reduction and internal fixation (CRIF) with DHS+DS. In addition, the biomechanical mechanism behind it was further analyzed with finite element analysis (FEA).

## 2. Methods

### 2.1. Patient Selection

This is a retrospective study that includes young patients with displaced FNF treated from January 2014 to August 2019 at the Third Hospital of Hebei Medical University. All patients received CRIF with DHS+DS. This study was performed in accordance with the STROBE guidelines and approved by the Institutional Review Board of the Third Hospital of Hebei Medical University. All patients in this study had written informed consent. Inclusion criteria were patients aged ≥18 years and ≤65 years, with isolated and displaced FNFs, and receiving CRIF with DHS+DS. However, the exclusion criteria were the following: (1) pathological fractures, (2) old fractures (>21 days after primary injury), and (3) reoperation for certain reasons. Patients with rheumatic disease and malignancies were also excluded. Finally, a total of 68 patients were included in our study.

### 2.2. Surgical Method and Postoperative Management

All surgeries were performed by senior surgeons with more than 15 years of clinical experience and under fluoroscopic guidance without percutaneous capsulotomy. Two grams of cefazolin was given intravenously 30 minutes preoperatively. After the anesthesia was significantly effective, the patient was positioned supine on a traction table with the unaffected limb in a semilithotomy position. Gradually increased traction was applied to disimpact the fracture. Under continuous traction, the affected limb was adducted to 40-45 degrees and internally rotated. The degree of internal rotation was individualized and case dependent. In case of difficulty to obtain anatomical reduction after two attempts, the positive buttress reduction (Gotfried reduction) was adopted for some patients [19], while anatomical reduction was continuously sought in other patients. When the satisfactory reduction was obtained, a straight lateral incision of proximal femur with approximate 10 cm was performed directly through the fascia to the bone. Then, two temporary guide pins were inserted into the femoral neck and head in order to obtain rotation stability. A single 6.5 mm cannulated derotational screw (Double Medical, Xiamen city, China) was inserted along the cranial guide pin, and one DHS lag screw (Double Medical, Xiamen city, China) of appropriate length was implanted along the caudal guide pin. Both screws should be entered up into the subchondral bone of the femoral head. Afterwards, a dynamic hip plate was fixed to the shaft with 2-4 screws. Finally, after reconfirming the reduction of fracture and position of internal fixation under the C-arm fluoroscopy, the incision was washed with normal saline and closed. Postoperatively, cefazolin (2 g × 3 doses) was administered intravenously for 24 hours. Quadriceps exercises were performed on the first day after surgery. Weight-bearing exercise was not allowed within 1 month after operation; then, rehabilitation guidance was implemented according to the outcomes of outpatient follow-up.

### 2.3. Radiographic Assessment

Plain radiographs of all patients at admission, immediately after surgery, and the last follow-up were analyzed by two experienced orthopedists who did not participate in the operation procedure. Anteroposterior radiographs (AP) were used to evaluate the level of fracture, the Garden classification, and the Pauwels classification. According to the Gotfried reduction method [16], all patients were categorized into the positive buttress reduction group (PBRG) and the anatomical reduction group (ARG).

### 2.4. Data Collection

Demographic, clinical, and surgical data were obtained from the patient medical record. Demographic data included age, gender, and affected side. Clinical information included injury mechanism, Garden grade, Pauwels classification, fracture level, time to surgery, and follow-up duration. Data on surgical procedure included the American Society of Anesthesiology (ASA) classification, surgical time, and fluoroscopy time.

### 2.5. Main Outcome Measurements

The main outcomes of this study were complications and clinical outcomes at the last follow-up. Complications included mechanical failure, fracture nonunion, and necrosis of the femoral head. The definition of fracture nonunion was the persistence of a fracture line over 8 months after the operation. The clinical outcomes included Harris hip score and visual analogue scale (VAS) at the last follow-up. According to previous studies [20], the Harris hip score was divided into four groups: excellent 90-100, good 80-89, fair 70-79, and poor <70. Additionally, the varus changes of the femoral neck shaft angle (FNSA) and the femoral neck shortening were also recorded. The FNSA was defined as the angle between the head-neck axis and the medullary axis of the shaft [10]. The method proposed by Zlowodzki et al. [21] was used to measure the length of the femoral neck.

### 2.6. Finite Element Analysis

**Figure 1:**
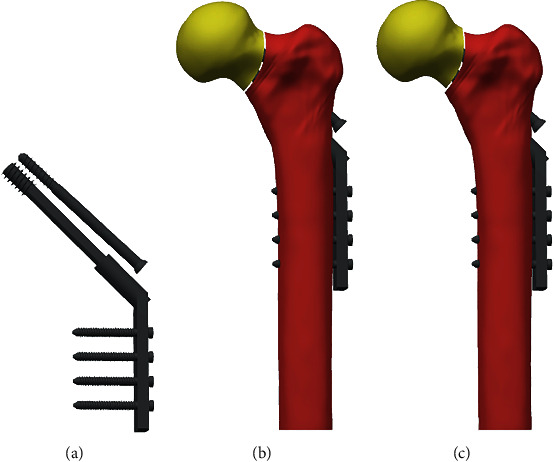
Finite element model. (a) DHS+DS model. (b) Anatomical reduction model. (c) Positive buttress reduction model.

After identifying risk factors for complications, we recruited a healthy Chinese woman (age: 59 years; body weight: 60 kg; height 158 cm) for a computed tomography (CT) scan (Sensation 64, Siemens Medical Solutions, Forchheim, Germany) to develop a FEA model (Figure 1). Scanning parameters included the following: scan slice thickness, 0.625 mm; scanning voltage, 120 kV; and scanning current, 300 mA. CT data of the left proximal femur were obtained and saved in Digital Imaging and Communications in Medicine (DICOM) format. Reconstruction of the proximal femoral model was performed using Mimics 20 (Materialise Technologies, Leuven, Belgium) and GeomagicStudio12 software (Raindrop Geomagic Inc., Morrisville, NC, USA). A Pauwels III FNF (Pauwels angle > 70°) was modeled using SolidWorks software (SolidWorks, Dassault Systèmes, USA). Meanwhile, the positive buttress reduction and the anatomical reduction were modeled. After that, DHS+DS was assembled into the bone. Ansys software (Ansys, Canonsburg, PA, USA) was used for mechanical analysis. The distal end of the femur was restrained. The properties of DHS+DS and bone were defined as linear elastic materials. The density of all the bones was calculated according to the Hu value of CT using the formula in previous studies [22]. For DHS+DS, we used values for titanium, which has Young's modulus (*E*) of 110,000 MPa and Poisson's ratio of 0.3 [23, 24]. Bone resorption at the fracture site was modeled by creating a 1 mm wide gap along the fracture plane [25, 26]. A load of 2100 N was applied above the femoral head. Ten points at the proximal fracture fragment were selected to measure their downward displacement. The distribution of von Mises stress, the peak stress of DHS+DS, and the displacement of the proximal fragment were recorded.

### 2.7. Statistical Analysis

Data were analyzed with SPSS 26 software (IBM Corp., Armonk, NY, USA). The interobserver reliability was evaluated with the kappa coefficient (*κ*) for Pauwels and Garden classifications, while intraclass correlation coefficient (ICC) for varus change of FNSA and femoral neck shortening. The Shapiro–Wilk test was used to evaluate the normality of continuous data. Categorical data were recorded as numbers (%) and evaluated with chi-squared or Fisher's exact test, where applicable. On the other hand, the continuous data were expressed as mean ± standard deviation (SD) and analyzed with the Student *t*-test or Mann–Whitney *U* test, as appropriate. Statistical significance was defined as *p* < 0.05.

## 3. Results

### 3.1. Patient Baseline Data

A total of 68 young patients with FNF were included in this study. The average duration of follow-up was 51.74 (22.0-89.0) months. Of all patients, 44 were men and 24 were women, with a mean age of 49.7 years (range 25-65). The interobserver reliability of radiographic characteristics was evaluated and is presented in Table 1. Patient characteristics are shown in Table 2. No significant difference was found between the two groups in age (*p* = 0.191), gender (*p* = 0.590), affected side (*p* = 0.701), injury mechanism (*p* = 0.223), Garden classification (*p* = 0.327), Pauwels classification (*p* = 0.764), fracture level (*p* = 0.642), time to surgery (*p* = 0.203), follow-up duration (*p* = 0.207), and ASA classification (*p* = 0.555). The mean operative time was nearly 19 minutes longer in the ARG (*p* = 0.022). Meanwhile, a shorter intraoperative fluoroscopy time was observed in the PBRG compared to the ARG (21.68 ± 8.53 vs. 28.11 ± 7.52, *p* = 0.002).

### 3.2. Clinical Outcomes and Complications

Most patients in both groups can return to normal life within six months after surgery, except for two patients in the ARG due to nonunion. Eight patients (11.8%) experienced at least one complication after surgery. There were no significant differences in necrosis of the femoral head (*p* = 0.681), fracture nonunion (*p* = 0.496), and mechanical failure (*p* = 0.620) between the two groups (Table 3). The degree of femoral neck varus was lower in the PBRG than in the ARG (1.95 ± 0.82° vs. 2.74 ± 1.25°, *p* = 0.03). Meanwhile, less femoral neck shortening was found in the PBRG compared to the ARG (2.06 ± 0.85 mm vs. 3.58 ± 1.26 mm, *p* < 0.001). The VAS score was similar between the two groups (*p* = 0.222). The Harris hip score did not differ between the two groups (*p* = 0.320). However, the PBRG showed a trend of higher excellent-good rate compared to the ARG (83.9% vs. 64.8%), although this was not statistically significant (*p* = 0.077) due to the limited sample size. After univariate analysis of the complications, age (*p* = 0.005) and gender (*p* = 0.019) were identified as risk factors for complications (Table 4).

### 3.3. Finite Element Analysis Outcomes

**Figure 2:**
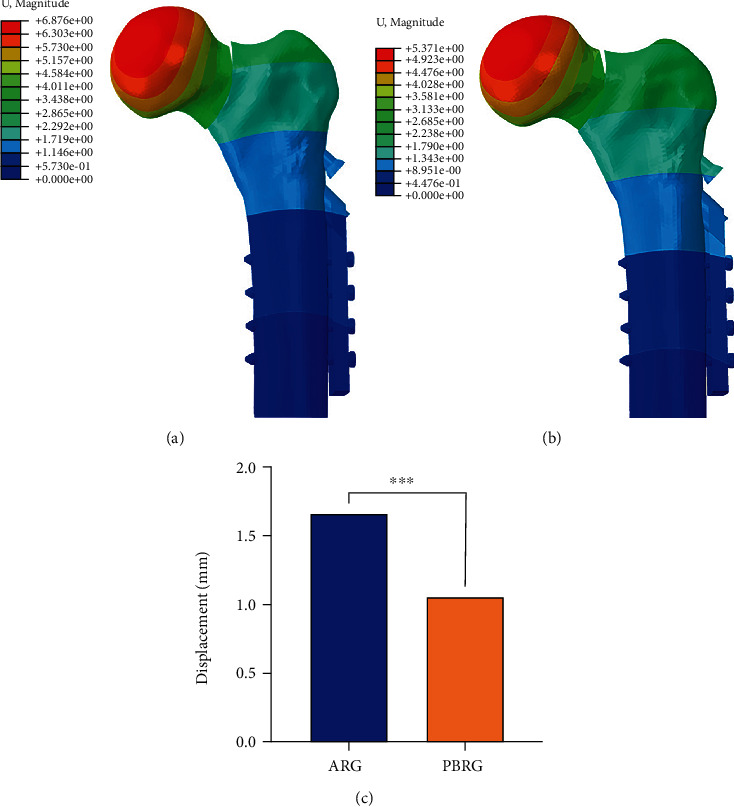
Displacement diagram of different types of reduction: (a) anatomical reduction; (b) positive buttress reduction; (c) comparison of the motion of the proximal femoral neck fragment motion (mm) for the two types of reduction.

**Figure 3:**
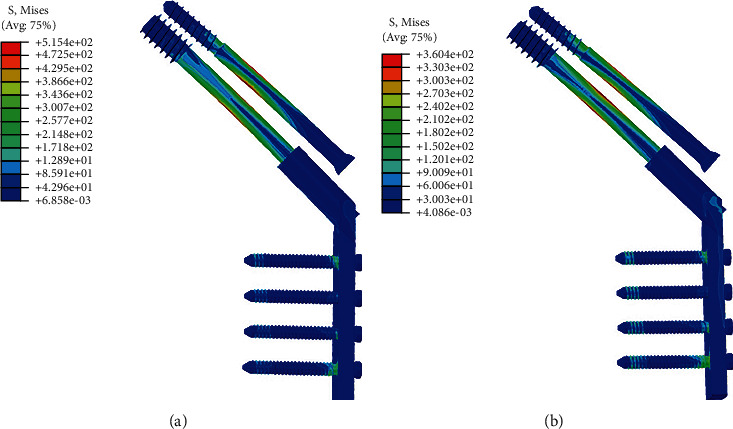
The von Mises stress distribution of DHS+CS in anatomical reduction (a) and positive buttress reduction (b).

It is worth mentioning that an anatomical reduction can develop to a negative buttress position in our FEA model. The displacement map showed that less downward movement was observed after the positive buttress reduction compared to the anatomical reduction (Figure 2). Furthermore, anatomical reduction had significantly greater implant stress (515 MPa) than positive buttress reduction (360 MPa) (Figure 3).

## 4. Discussion

Successful reduction and firm fixation to obtain sufficient stability are vital techniques for fixing displaced FNFs in young patients. Despite recent improvements in implant design, DHS+DS was still one of the best choices for the treatment of displaced FNFs [27]. Furthermore, many studies on FNFs treated with DHS+CS have included reduction quality, but only as a confounding variable [28–30]. To our knowledge, the present study was the first study to focus on biomechanical and clinical results of different qualities of reduction in the treatment of FNFs with internal fixation of DHS+DS. The results demonstrated that a higher good-excellent rate of the Harris hip score could be obtained through positive buttress reduction compared to anatomical reduction. In addition, the degree of femoral neck shortening and varus change of the FNSA was lower after positive buttress reduction than anatomical reduction.

Due to adverse clinical outcomes [31–33], the treatments of displaced FNFs in young patients are still a challenge for orthopedists. Although multiple cannulated screws may effectively manage most of displaced FNFs, DHS was recommended for vertical and highly comminuted unstable fractures [27, 34]. The addition of a derotational screw has been shown to improve the mechanical stability of FNFs [35, 36]. In a biomechanical study, Samsami et al. [37] demonstrated that there was a significant difference between DHS+DS and CSS in axial femoral head displacement (0.94 mm vs. 2.3 mm) and average displacement of fracture fragments (1.5 *μ*m vs. 70 *μ*m), which indicated that DHS+DS had a more stable fixation than CCS for FNFs. In our study, the rate of femoral head necrosis (8.8%) and fracture nonunion (2.9%) was lower compared to historical series using CCS alone [10, 14, 17]. Our findings showed that DHS+DS was a more effective choice for FNFs in young patients compared with CCS.

**Figure 4:**
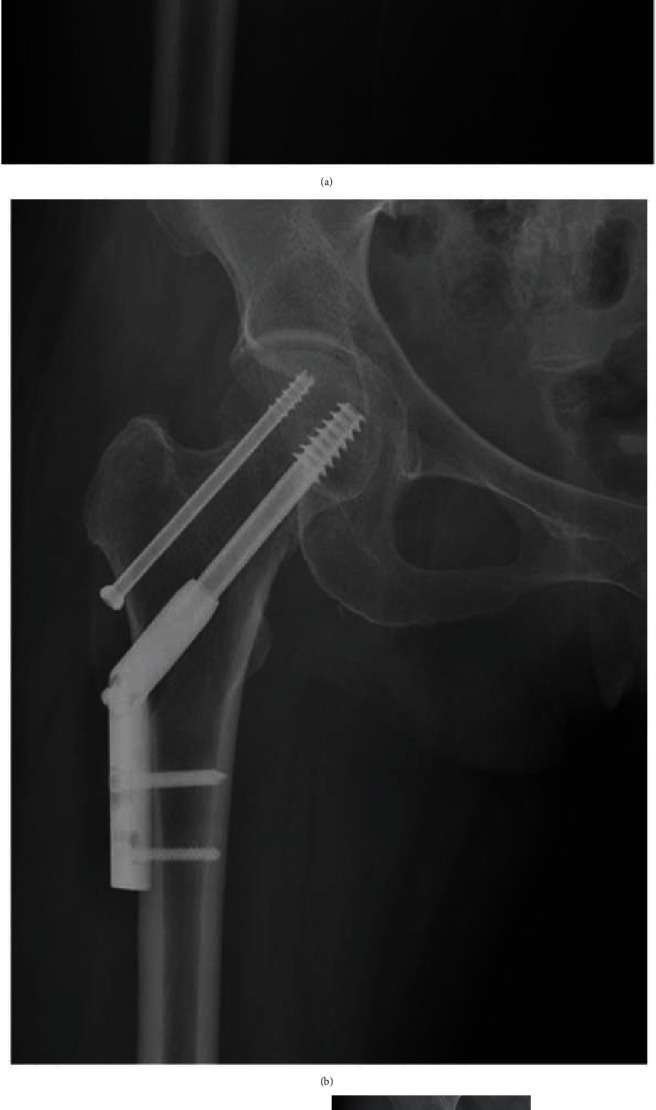
A 59-year-old female patient with FNF. (a) Preoperative radiograph showing a Pauwels III type FNF. (b) Radiograph immediately after surgery showing an anatomical reduction was achieved. (c) Radiograph at six weeks postoperatively showing the anatomical reduction converted to a negative buttress position. (d) Postoperative view at 12 months showing nonunion of the fracture. (e) Radiograph at 20 months postoperatively showing femoral head necrosis. (f) Radiographs at 24 months after surgery showing that a total hip replacement was performed.

**Figure 5:**
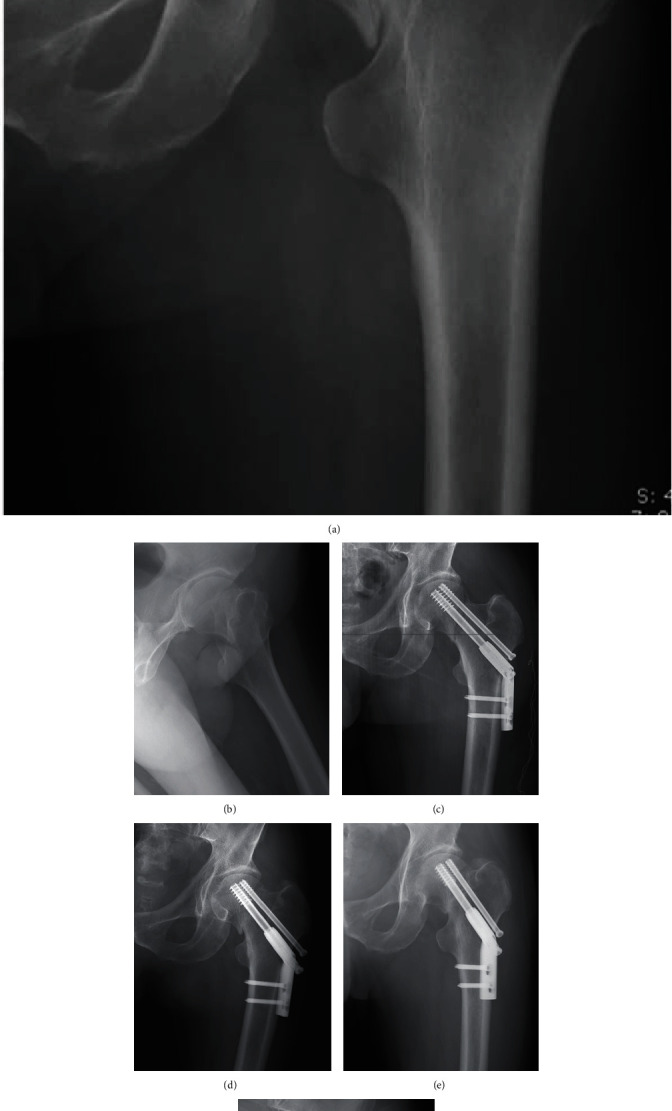
Follow-up of a 63-year-old male patient with FNF. (a) Preoperative AP radiograph. (b) Preoperative lateral radiograph. (c) Radiograph immediately after surgery showing an anatomical reduction of fracture. (d) Radiograph at 4 weeks postoperatively showing the anatomical reduction changed to negative buttress position. (e) AP radiograph at 6 months postoperatively showing femoral neck shortening. (f) Radiograph at 24 months after surgery showing the femoral neck fracture healed in a deformed position.

For FNF in young patients, anatomical reduction and firm fixation are the main treatment choice, particularly in those with abundant daily activities. A satisfactory reduction can offer maximum contact between fracture fragments, improve internal fixation stability, and thus promote fracture healing. Therefore, the anatomical reduction of FNF has been repeatedly emphasized in previous literature. However, it can be a formidable task to achieve this goal [10, 11]. Due to difficulty of achieving reduction in our cohort, more attempts were needed to obtain an anatomical reduction in ARG compared with the PBRG, which significantly increased surgical time, leading to excessive radiation exposure. As a result, new damage at the fracture site may occur, which can increase the incidence of nonunion and avascular necrosis of the femoral head. Similar results were also reported in previous studies. Zhao et al. [14] observed that patients with positive reduction had a lower proportion of reoperation than those patients with anatomical reduction (8.6% vs. 12.2%). Chua et al. [25] reported that the difficulty of achieving reduction was an important predictor of internal fixation failure in displaced FNF. In our study, although there was no significant difference between the two groups in postoperative complications, a higher good-excellent Harris hip score rate was observed in the PBRG. Moreover, it was worth mentioning that even if an anatomic reduction of FNF was obtained intraoperatively, a redisplacement may occur postoperatively (Figures 4 and 5). After comparing the groups with and without complications, we found that female and older age were risk factors for postoperative complications, which was similar to a previous study [38]. By using the FEA with a proximal femur of a 59-year-old female, we testified that an initial anatomical reduction could develop to a negative buttress position in the presence of a fracture gap and Pauwels III fracture. We consider that postmenopausal osteoporosis may be the main reason for this phenomenon. Therefore, based on our findings, we suggest that anatomical reduction may not always be the best option for young FNF patients with difficulty of reduction, especially in postmenopausal females.

**Figure 6:**
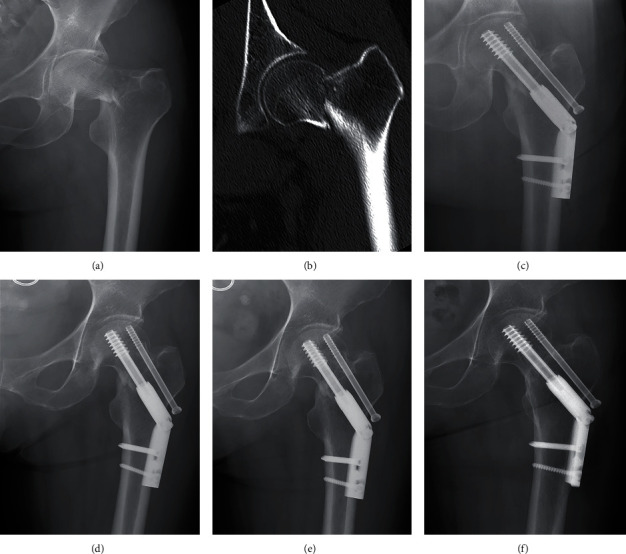
Typical case of positive buttress reduction (female, 52-year-old). (a) Preoperative AP radiograph. (b) CT scan of the same patient. (c) Radiograph immediately after surgery showing positive buttress reduction. (d–f) Radiographs at 1 month, 3 months, and 12 months of follow-up: no complication occurred.

When FNF occurs, the medial femoral circumflex artery is always injured, which can result in intra-articular hemorrhage and increase pressure in the joint capsule [39]. As a result, it is not easy to achieve an anatomical reduction via CRIF. In our cohort, approximately 46% of FNF were reduced in a nonanatomical position, which was consistent with previous studies [10, 14]. However, none of the patients in the PBRG developed to a negative buttress position during follow-up (Figure 6). This means that positive buttress reduction can reduce the negative influence of bone resorption at the fracture site. After bone resorption occurs at the fracture site, the inferior cortex of the proximal fragment will be supported by the calcar cortex of the distal fragment during the process of downward displacement. Its function was similar to a medial buttress plate [14]. This could be the main reason why in our study, the PBRG had a lower degree of femoral neck shortening and varus than the ARG. The cortex-to-cortex buttress combined with DHS+DS would provide a sustainable stability environment for fracture union and produce predictable clinical outcomes. In addition, positive buttress reduction can improve the FNF repairment through enhancing osteogenesis and angiogenesis. In a study focused on histological reconstruction and biomechanics, Wang et al. [40] found that positive buttress reduction of FNFs can upregulate the expression of bone morphogenetic protein-2 (BMP2) and angiopoietin (ANGPT) and thus promote bone and blood vessel formation. Our findings are helpful for guiding the reduction of FNFs during operation. If a secondary redisplacement is anticipated in the FNF operation, a reduction in the positive buttress position may be considered.

## 5. Limitations

Our study suffers from several limitations. First, this study was a retrospective design and included a relatively small number of cases. Prospective studies and larger samples are needed in the future to draw more reliable conclusions. Second, the operations were performed by multiple surgeons, which may bias the surgical outcomes to a certain extent. Third, as with other finite element analyses, we simplified the bone model from the anisotropic viscoelastic material to uniform material properties, and the fracture plane from rough with interdigitation to a smooth surface with friction. Future model construction could improve the analysis by using a more realistic facture morphology and bone properties.

## 6. Conclusion

For displaced FNF with difficulty to achieve reduction, DHS+DS combined with positive buttress reduction was an effective treatment in young patients due to better mechanical support, shorter surgical time, less radiation exposure, and higher excellent-good rate of Harris hip score.

## Figures and Tables

**Table 1:** Interobserver reliability of the radiographic characteristics.

Characteristics	ICC or *k*	95% CI	*p* value
Pauwels classification, *κ*	0.798	0.608 to 0.988	<0.001^∗^
Garden classification, *κ*	0.837	0.682 to 0.992	<0.001^∗^
Femora neck shortening, ICC	0.916	0.868 to 0.947	<0.001^∗^
Femoral neck varus, ICC	0.889	0.826 to 0.930	<0.001^∗^

Abbreviations: CI: confidence interval; ICC: intraclass correlation coefficient; *κ*: kappa coefficient. ^∗^Statistically significant difference.

**Table 2:** Baseline characteristics of the included patients with different qualities of reduction.

Characteristics	Positive buttress reduction group (*n* = 31)	Anatomical reduction group (*n* = 37)	*p* value
Age (years)	47.71 ± 12.32	51.32 ± 10.24	0.191
Gender			0.590
Female	12 (38.7)	12 (32.4)	
Male	19 (61.3)	25 (67.6)	
Affected side			0.701
Right	14 (45.2)	15 (40.5)	
Left	17 (54.8)	22 (59.5)	
Injury mechanism			0.223
Fall injuries	18 (58.1)	16 (43.2)	
Traffic accident injuries	13 (41.9)	21 (56.8)	
Garden classification			0.327
Type III	22 (71.0)	30 (81.1)	
Type IV	9 (29.0)	7 (18.9)	
Pauwels classification			0.764
II	5 (16.1)	7 (18.9)	
III	26 (83.9)	30 (81.1)	
Fracture level			0.642
Subcapital	19 (61.3)	18 (48.6)	
Midcapital	11 (35.5)	17 (45.9)	
Basicvervical	1 (3.2)	2 (5.4)	
Time to surgery (days)	5.19 ± 1.96	5.81 ± 1.99	0.203
Follow-up duration (months)	47.81 ± 22.22	55.03 ± 24.10	0.207
ASA classification	1.3 ± 0.54	1.41 ± 0.60	0.555
Surgical time (minutes)	120.97 ± 32.60	139..86 ± 33.26	0.022^∗^
Fluoroscopy time (minutes)	21.68 ± 8.53	28.11 ± 7.52	0.002^∗^

Abbreviation: ASA: American Society of Anesthesiology classification. ^∗^Statistically significant difference.

**Table 3:** Comparison of postoperative complications and clinical outcomes.

Variables	Positive buttress reduction group (*n* = 31)	Anatomical reduction group (*n* = 37)	*p* value
Complications			
Varus change of the FNSA (degree)	1.95 ± 0.82	2.74 ± 1.25	0.003^∗^
Femoral neck shortening (mm)	2.06 ± 0.85	3.58 ± 1.26	<0.001^∗^
Femoral head necrosis	2 (6.5)	4 (10.8)	0.681
Fracture nonunion	0 (0.0)	2 (5.4)	0.496
Mechanical failure	1 (3.2)	3 (8.1)	0.620
VAS	1.97 ± 0.84	2.22 ± 0.82	0.222
Harris hip score			0.320
Excellent	8 (25.8)	6 (16.2)	
Good	18 (58.1)	18 (48.6)	
Fair	4 (12.9)	9 (24.3)	
Poor	1 (3.2)	4 (10.8)	

Abbreviations: FNSA: femoral neck-shaft angle; VAS: visual analogue scale. ^∗^Statistically significant difference.

**Table 4:** Univariate analysis to evaluate the risk factor for complications.

Variables	Without complications (*n* = 60)	With complications (*n* = 8)	*p* value
Age (years)	48.57 ± 11.35	58.00 ± 6.74	0.005^∗^
Gender			0.019^∗^
Female	18 (30.0)	6 (75.0)	
Male	42 (70.0)	2 (25.0)	
Affected side			0.451
Right	27 (45.0)	2 (25.0)	
Left	33 (55.0)	6 (75.0)	
Injury mechanism			1.000
Fall injuries	30 (50.0)	4 (50.0)	
Traffic accident injuries	30 (50.0)	4 (50.0)	
Garden type			0.670
Type III	45 (75.0)	7 (87.5)	
Type IV	15 (25.0)	1 (12.5)	
Pauwels classification			0.334
II	12 (20.0)	0 (0.0)	
III	48 (80.0)	8 (100.0)	
Fracture level			0.402
Subcapital	33 (55.0)	4 (50.0)	
Midcapital	25 (41.7)	3 (41.7)	
Basicvervical	2 (3.3)	1 (3.3)	
ASA classification	1.32 ± 0.50	1.75 ± 0.89	0.215
Time to surgery (days)	5.67 ± 1.97	4.50 ± 1.85	0.118
Follow-up duration (months)	52.55 ± 23.64	45.63 ± 21.69	0.435

Abbreviations: ASA: American Society of Anesthesiology classification. ^∗^Statistically significant difference.

## Data Availability

The data used to support the findings of this study are available from the corresponding authors upon request.

## Abbreviations

FNFs: Femoral neck fractures
DHS: Dynamic hip screw
DS: Derotation screw
CCS: Cannulated screw system
CRIF: Closed reduction and internal fixation
FEA: Finite element analysis
AP: Anteroposterior
PBRG: Positive buttress reduction group
ARG: Anatomical reduction group
ASA: American Society of Anesthesiology
VAS: Visual analogue scale
FNSA: Femoral neck-shaft angle
CT: Computed tomography
DICOM: Digital Imaging and Communications in Medicine
*κ*: Kappa coefficient
ICC: Intraclass correlation coefficient
SD: Standard deviation
BMP: Bone morphogenetic protein-2
ANGPT: Angiopoietin.

## References

[1] Thorngren K. G., Hommel A., Norrman P. O., Thorngren J., Wingstrand H. (2002). Epidemiology of femoral neck fractures. *Injury*.

[2] Bhandari M., Swiontkowski M. (2017). Management of acute hip fracture. *The New England Journal of Medicine*.

[3] Ly T. V., Swiontkowski M. F. (2008). Treatment of femoral neck fractures in young adults. *The Journal of Bone and Joint Surgery. American Volume*.

[4] Bonnaire F. A., Weber A. T. (2002). Analysis of fracture gap changes, dynamic and static stability of different osteosynthetic procedures in the femoral neck. *Injury*.

[5] Massoud E. I. (2010). Fixation of basicervical and related fractures. *International Orthopaedics*.

[6] Sağlam N., Küçükdurmaz F., Kivilcim H., Kurtulmuş T., Sen C., Akpinar F. (2014). Biomechanical comparison of antirotator compression hip screw and cannulated screw fixations in the femoral neck fractures. *Acta Orthopaedica et Traumatologica Turcica*.

[7] Aminian A., Gao F., Fedoriw W. W., Zhang L. Q., Kalainov D. M., Merk B. R. (2007). Vertically oriented femoral neck fractures: mechanical analysis of four fixation techniques. *Journal of Orthopaedic Trauma*.

[8] Deneka D. A., Simonian P. T., Stankewich C. J., Eckert D., Chapman J. R., Tencer A. F. (1997). Biomechanical comparison of internal fixation techniques for the treatment of unstable basicervical femoral neck fractures. *Journal of Orthopaedic Trauma*.

[9] Freitas A., Toledo Júnior J. V., Ferreira Dos Santos A., Aquino R. J., Leão V. N., Péricles de Alcântara W. (2020). Biomechanical study of different internal fixations in Pauwels type III femoral neck fracture - a finite elements analysis. *J Clin Orthop Trauma.*.

[10] Xiong W. F., Chang S. M., Zhang Y. Q., Hu S. J., Du S. C. (2019). Inferior calcar buttress reduction pattern for displaced femoral neck fractures in young adults: a preliminary report and an effective alternative. *Journal of Orthopaedic Surgery and Research*.

[11] Zhuang L., Wang L., Xu D., Wang Z. (2019). Anteromedial femoral neck plate with cannulated screws for the treatment of irreducible displaced femoral neck fracture in young patients: a preliminary study. *European Journal of Trauma and Emergency Surgery*.

[12] Su Y., Chen W., Zhang Q. (2011). An irreducible variant of femoral neck fracture: a minimally traumatic reduction technique. *Injury*.

[13] Wang G., Wang B., Tang Y., Yang H. L. (2019). A quantitative biomechanical study of positive buttress techniques for femoral neck fractures: a finite element analysis. *Chinese Medical Journal (Engl)*.

[14] Zhao G., Liu C., Chen K. (2021). Nonanatomical reduction of femoral neck fractures in young patients (≤65 years old) with internal fixation using three parallel cannulated screws. *BioMed Research International*.

[15] Zhao G., Liu M., Li B., Sun H., Wei B. (2021). Clinical observation and finite element analysis of cannulated screw internal fixation in the treatment of femoral neck fracture based on different reduction quality. *Journal of Orthopaedic Surgery and Research*.

[16] Gotfried Y., Kovalenko S., Fuchs D. (2013). Nonanatomical reduction of displaced subcapital femoral fractures (Gotfried reduction). *Journal of Orthopaedic Trauma*.

[17] Huang K., Fang X., Li G., Yue J. (2020). Assessing the effect of Gotfried reduction with positive buttress pattern in the young femoral neck fracture. *Journal of Orthopaedic Surgery and Research*.

[18] Zhang Y. Q., Chang S. M. (2013). Mechanism of "Gotfried reduction" in femoral neck fracture. *Journal of Orthopaedic Trauma*.

[19] Gotfried Y. (2012). The Gotfried (Nonanatomic, closed) reduction of unstable subcapital femoral fractures. *Techniques in Orthopaedics*.

[20] Harris W. H. (1969). Traumatic arthritis of the hip after dislocation and acetabular fractures: treatment by mold arthroplasty. An end-result study using a new method of result evaluation. *The Journal of Bone and Joint Surgery. American Volume*.

[21] Zlowodzki M., Brink O., Switzer J. (2008). The effect of shortening and varus collapse of the femoral neck on function after fixation of intracapsular fracture of the hip. *The Journal of Bone and Joint Surgery. British volume*.

[22] Reina-Romo E., Rodríguez-Vallés J., Sanz-Herrera J. A. (2018). In silico dynamic characterization of the femur: physiological versus mechanical boundary conditions. *Medical Engineering & Physics*.

[23] Benli S., Aksoy S., Havitcioğlu H., Kucuk M. (2008). Evaluation of bone plate with low-stiffness material in terms of stress distribution. *Journal of Biomechanics*.

[24] Fan Y., Xiu K., Duan H., Zhang M. (2008). Biomechanical and histological evaluation of the application of biodegradable poly-L-lactic cushion to the plate internal fixation for bone fracture healing. *Clinical Biomechanics (Bristol, Avon)*.

[25] Chua D., Jaglal S. B., Schatzker J. (1998). Predictors of early failure of fixation in the treatment of displaced subcapital hip fractures. *Journal of Orthopaedic Trauma*.

[26] Cordeiro M., Caskey S., Frank C., Martin S., Srivastava A., Atkinson T. (2020). Hybrid triad provides fracture plane stability in a computational model of a Pauwels type III hip fracture. *Computer Methods in Biomechanics and Biomedical Engineering*.

[27] Florschutz A. V., Langford J. R., Haidukewych G. J., Koval K. J. (2015). Femoral neck Fractures. *Journal of Orthopaedic Trauma*.

[28] Chen C., Yu L., Tang X. (2017). Dynamic hip system blade versus cannulated compression screw for the treatment of femoral neck fractures: a retrospective study. *Acta Orthopaedica et Traumatologica Turcica*.

[29] Duffin M., Pilson H. T. (2019). Technologies for young femoral neck fracture fixation. *Journal of Orthopaedic Trauma*.

[30] Jacob G., Pai S., Huggi V. (2020). Lag screw with DHS (LSD) for vertical angle femoral neck fractures in young adults. *Injury*.

[31] Slobogean G. P., Sprague S. A., Scott T., Bhandari M. (2015). Complications following young femoral neck fractures. *Injury*.

[32] Slobogean G. P., Stockton D. J., Zeng B. F., Wang D., Ma B., Pollak A. N. (2017). Femoral neck shortening in adult patients under the age of 55 years is associated with worse functional outcomes: Analysis of the prospective multi- center study of hip fracture outcomes in China (SHOC). *Injury*.

[33] Stockton D. J., Lefaivre K. A., Deakin D. E. (2015). Incidence, magnitude, and predictors of shortening in young femoral neck fractures. *Journal of Orthopaedic Trauma*.

[34] Makki D., Mohamed A. M., Gadiyar R., Patterson M. (2013). Addition of an anti-rotation screw to the dynamic hip screw for femoral neck fractures. *Orthopedics*.

[35] Kemker B., Magone K., Owen J., Atkinson P., Martin S., Atkinson T. (2017). A sliding hip screw augmented with 2 screws is biomechanically similar to an inverted triad of cannulated screws in repair of a Pauwels type-III fracture. *Injury*.

[36] Johnson J. P., Borenstein T. R., Waryasz G. R. (2017). Vertically oriented femoral neck fractures: a biomechanical comparison of 3 fixation constructs. *Journal of Orthopaedic Trauma*.

[37] Samsami S., Augat P., Rouhi G. (2019). Stability of femoral neck fracture fixation: a finite element analysis. *Proceedings of the Institution of Mechanical Engineers. Part H*.

[38] Ramadanov N., Toma I., Herkner H., Klein R., Behringer W., Matthes G. (2020). Factors that influence the complications and outcomes of femoral neck fractures treated by cannulated screw fixation. *Scientific Reports*.

[39] Firoozabadi R. (2020). CORR insights®: does screw location affect the risk of subtrochanteric femur fracture after femoral neck fixation? A biomechanical study. *Clinical Orthopaedics and Related Research*.

[40] Wang G., Wang B., Wu X., Yang H. (2020). Gotfried positive reduction promotes the repair of femoral neck fracture potentially via enhancing osteogenesis and angiogenesis. *Biomedicine & Pharmacotherapy*.

